# Reduced intestinal butyrate availability is associated with the vascular remodeling in resistance arteries of hypertensive rats

**DOI:** 10.3389/fphys.2022.998362

**Published:** 2022-09-29

**Authors:** Patrizia Dardi, Rosangela Aparecida dos Santos-Eichler, Sarah de Oliveira, Marco Aurélio Ramirez Vinolo, Niels Olsen Saraiva Câmara, Luciana Venturini Rossoni

**Affiliations:** ^1^ Laboratory of Vascular Physiology, Institute of Biomedical Science, Department of Physiology and Biophysics, University of Sao Paulo, Sao Paulo, Brazil; ^2^ Laboratory of Cellular Communication, Institute of Biomedical Science, Department of Pharmacology, University of Sao Paulo, Sao Paulo, Brazil; ^3^ Laboratory of Immunoinflammation, Institute of Biology, Department of Genetics, Evolution, Microbiology and Immunology, University of Campinas, Campinas, Brazil; ^4^ Laboratory of Transplantation Immunobiology, Institute of Biomedical Science, Department of Immunology, University of Sao Paulo, Sao Paulo, Brazil

**Keywords:** hypertension, resistance arteries, vascular remodeling, short-chain fatty acids, butyrate, SCFAs-sensing receptors

## Abstract

During hypertension an unbalance of short-chain fatty acids (SCFAs) production by intestinal bacteria is described. However, no data evaluate the association of SCFAs and vascular remodeling in hypertension, which is an important hallmark of this disease. Thus, the present study aims to evaluate the correlations between SCFAs availability and the resistance arteries remodeling in hypertension, as well as to identify the possible pathway by which the SCFAs could exert a structural and mechanical influence. Hence, male spontaneously hypertensive rats (SHR) and normotensive Wistar rats had blood pressure measured by tail-cuff plethysmography; fecal SCFAs content assessed by gas chromatography; gene expression of SCFAs-transporters in gut epithelium and SCFAs-sensing receptors on mesenteric resistance arteries (MRA) quantified by PCR; and MRA structural and mechanical parameters analyzed by pressure myograph. Reduced butyrate fecal content was found in SHR, with no changes in propionate and acetate, as well as decreased mRNA levels of SCFAs-transporters (MCT1, MCT4, and SMCT1) in the intestinal epithelium. In addition, lower gene expression of SCFAs-sensing receptors (GPR41, GPR43, and GPR109a, but not Olfr78) was identified in MRAs of SHR, which also shows inward eutrophic remodeling with stiffness. Butyrate content presented a negative correlation with systolic blood pressure and with the structural alterations found on MRAs, while a positive correlation between butyrate content and mechanical parameters was detected. Altogether the present study suggests that lower butyrate content due to ineffective SCFA bioavailability, associated with lower SCFAs-sensing receptors expression, could favor MRA remodeling, increasing peripheral vascular resistance and worsening hypertension prognosis.

## 1 Introduction

Since the 1980s, researchers have been trying to decipher the short-chain fatty acids (SCFAs) role in the regulation of vascular homeostasis (Miller et al., 1981; Daugirdas and Nawab, 1987; Mortensen et al., 1990; Nutting et al., 1991; Nutting et al., 1992; Mortensen et al., 1994; Aaronson et al., 1996; Knock et al., 2002). It is well known that the main SCFAs (acetate, propionate, and butyrate) are produced from the fermentation of complex carbohydrates, by anaerobic bacteria present in the large intestine (Cummings, 1983). However, despite studies showing SCFAs promote vascular relaxation on different vascular beds (Miller et al., 1981; Daugirdas and Nawab, 1987; Mortensen et al., 1990; Nutting et al., 1991; Nutting et al., 1992; Mortensen et al., 1994; Aaronson et al., 1996; Knock et al., 2002), the role of those bacterial products on vascular remodeling is still unknown. Interestingly, in one of the few publications studying mesenteric resistance arteries (MRAs) structural and mechanical properties and microbiota composition, the lack of intestinal microbiota in male germ-free mice was correlated with inward hypotrophic remodeling and arterial stiffness (Edwards et al., 2020).

Vascular remodeling is considered a hallmark of hypertension (Mulvany, 2002; Masi et al., 2020), and this risk factor for cardiovascular disease has also been associated with intestinal microbiota dysbiosis in recent years (Yang et al., 2015; Adnan et al., 2017; Li et al., 2017; Kim et al., 2018; Toral et al., 2019; Felizardo et al., 2019; Silveira-Nunes et al., 2020; Robles-Vera et al., 2021). In spontaneously hypertensive rats (SHR) the presence of gut dysbiosis was identified, in addition to reduced butyrate and acetate-producing bacteria (Yang et al., 2015; Robles-Vera et al., 2020; Robles‐Vera et al., 2020), and smaller butyrate levels in fecal, plasma and serum samples (Wu et al., 2019; Yang et al., 2019; Robles‐Vera et al., 2020). When treated with butyrate for 13 weeks, SHR exhibited reduced systolic blood pressure and enhanced endothelium-dependent vasodilation in a conductance artery (thoracic aorta) as compared with untreated SHR (Robles‐Vera et al., 2020) and remarkably, those results were also observed when SHR were treated with different probiotic strains (Gómez-Guzmán et al., 2015; Robles‐Vera et al., 2020). With the available data, studies suggest that the influence of SCFAs on cardiovascular function could occur indirectly, acting on central cardio regulatory sites or on intestinal features (Toral et al., 2019a; Onyszkiewicz et al., 2019; Yang et al., 2019; Robles-Vera et al., 2020; Robles‐Vera et al., 2020); or even by modulation of vascular homeostasis in a direct way (Pluznick et al., 2013; Natarajan et al., 2016; Bartolomaeus et al., 2019; Poll et al., 2020). Moreover, we still have a long way ahead to finally comprehend the role of the SCFAs in vascular homeostasis and how their absence can favor the vascular hallmarks of hypertension.

Although some studies focused on the evaluation of the endothelial function of conductance vessels (Gómez-Guzmán et al., 2015; Karbach et al., 2016; Natarajan et al., 2016; Kim et al., 2018; Bartolomaeus et al., 2019; Toral et al., 2019b; Robles‐Vera et al., 2020), no published data assessed the influence of SCFAs on the structural and mechanical remodeling found in resistance arteries, which are pivotal to the control of peripheral vascular resistance and blood pressure regulation (Mulvany, 2002). Hence, the present study considers the hypothesis that there are correlations between butyrate availability and the vascular remodeling of SHR MRAs. To prove our hypothesis, we will evaluate the participation of SCFAs-transporters in gut epithelium and SCFAs-sensing receptors on MRAs, as a possible pathway by which the SCFAs could exert their structural and mechanical influence.

## 2 Materials and methods

### 2.1 Experimental groups

All experimental procedures were approved by the Animal Care and Use Committee of the Institute of Biomedical Sciences of the University of Sao Paulo (no. 1946260318) and were conducted in accordance with the Brazilian Council of Animal Research (CONCEA). All methods described here follow the Guidelines for Transparency on Gut Microbiome Studies in Essential and Experimental hypertension (Marques et al., 2019). Four-week-old male rats from Wistar and SHR strains were obtained from colonies of SPF rats at the production facility of the Institute of Biomedical Science of the University of Sao Paulo. SHR was chosen as a model of essential hypertension and Wistar rats were selected instead of Wistar-Kyoto as a normotensive control, once they possess lower blood pressure values, a more stable cardiac function, and distant genetic background compared to the hypertensive strain studied (Louis and Howes, 1990; Rezende et al., 2022). Rats were housed in groups of 3 animals per cage within each strain and were maintained in an animal house facility until they reached 6 months of age (25–27 weeks old). During this period, rats were kept under standard conditions of room temperature and a 12-h light/dark cycle, with free access to water and standard chow (Nuvilab, Quimtia, Argentina, with 220 g/kg of protein and 70 g/kg of fiber in composition).

### 2.2 Blood pressure measurement

Systolic blood pressure (SBP) measurements were performed by tail-cuff plethysmography (LE 5001, Panlabs. I. Barcelona, Spain) in lightly restrained, non-anesthetized rats. Animals were adapted to the equipment and the procedure for 2 days before the final measurement. At least 20 measurements were made at each session, and the mean was used to obtain the SBP level.

Animals were anesthetized with a mixture of ketamine and xylazine (90 and 10 mg/kg, *i. p.*, respectively; Sespo Indústria e Comércio, Paulínia, SP, Brazil) and were killed by exsanguination. Fecal samples obtained from the cecum and clean segments of the proximal colon were promptly collected and stored at -80°C, whilst the mesenteric arcade was carefully removed, placed in cold gassed (95% O_2_ and 5% CO_2_) Krebs-Henseleit solution (KHS) (in mmol/L: 118 NaCl, 4.7 KCl, 2.5 CaCl_2_•2H_2_O, 1.2 KH_2_PO_4_, 1.2 MgSO_4_•7H_2_O, 25 NaHCO_3_, 11 glucose and 0.01 EDTA, pH 7.4) and employed in the experimental protocols described below.

### 2.3 Pressure myograph

Structural and mechanical properties of third-order branch mesenteric resistance arteries (MRA) were evaluated in a pressure myograph (DMT, 115FP model, Aarhus, Denmark). MRA was dissected, freed of connective tissue, and maintained in cold-gassed KHS. Segments (∼6 mm in length) were cannulated into glass micropipettes, in the microvessel chamber, being held with nylon thread, in gassed KHS (pH 7.4, 37°C). Intraluminal pressure was raised to 140 mmHg to adjust the longitudinal artery stretch and to evaluate possible leakage. Then, intraluminal pressure was reduced to 70 mmHg, allowing the MRA segment to stabilize for 1 h. After this period, MRA was washed with gassed Ca^2+^ - free KHS (containing 10 mmol/L EGTA), being kept in this solution for 15 min to ensure Ca^2+^ depletion and complete relaxation. To confirm Ca^2+^ depletion, arteries were exposed to a high-potassium solution (120 mmol/L), certifying the absence of contractile response. After another washing and 15 min of stabilization in Ca^2+^ - free KHS, intraluminal pressure was reduced to 3 mmHg, initiating the pressure-diameter curve in a passive condition – performed by intraluminal pressure increments from 3 to 140 mmHg, with a 5-min interval at each intraluminal pressure step.

Internal and external diameters (Di_0Ca_, De_0Ca_) were continuously assessed by MyoView II software (DMT, Aarhus, Denmark) during the entire pressure-diameter curve. From the Di_0Ca_ and De_0Ca_ obtained values, the following structural and mechanical parameters were calculated:

$$Wall\;thickness\;\left(WT\right)=\left({\text{De}}_{0\text{Ca}}{-\text{Di}}_{0\text{Ca}}\right)/2$$

$$Wall/lumen\;ratio\;\left(W/L\right)\;=\left({\text{De}}_{0\text{Ca}}-{\text{Di}}_{0\text{Ca}}\right)/2x{\text{Di}}_{0\text{Ca}}$$

$$Cross-sectional\;area\;\left(CSA\right)=\left(\Pi /4\right)\;x\;({\mathrm{De}}_{0\text{Ca}}^{2}-{\mathrm{Di}}_{0\text{Ca}}^{2})$$

$$Incremental\;distensibility=\text{Δ}{\text{Di}}_{0\text{Ca}}/\;\left({\text{Di}}_{0\text{Ca}}\;x\;\Delta P\right)\;x\;100$$

$$Circumferential\;wall\;stress=\left(P\;x\;{\text{Di}}_{0\text{Ca}}\right)/\left(2\text{WT}\right)$$

$$Circumferential\;wall\;strain=\left({\text{Di}}_{0\text{Ca}}{-D}_{00\text{Ca}}\right)/D_{00\text{Ca}}$$

Incremental distensibility is a mechanical parameter that represents the percentage of distensibility change suffered by the arterial Di_0Ca_ for every mmHg change in the intraluminal pressure. To calculate circumferential wall stress values, another mechanical parameter, *P* represents the intraluminal pressure (1 mmHg = 1.334 x 10^3^ dyn•cm^−2^) and WT is the wall thickness at each intraluminal pressure, measured in the passive condition. For circumferential wall strain assessment, D_00Ca_ is the Di at 3 mmHg and Di_0Ca_ represents the internal diameter found in each given intraluminal pressure, across the entire pressure-diameter curve. Arterial stiffness was determined by the Young elastic modulus (E) in the stress-strain curves. By determining the stress-strain curve slope, the incremental elastic modulus is calculated, providing a value considered a direct index of arterial stiffness, the β angle.

### 2.4 Gene expression

Total mRNA from proximal colon segments and MRA were obtained using Trizol (Invitrogen, Carlsbad, California, EUA) according to the manufacturer’s recommendations. For proximal colon segments 2 µg of mRNA was reverse transcribed using High-Capacity cDNA Reverse Transcription (Thermo Fisher, Waltham, MA, United States); whereas for MRA 5 µg of mRNA was employed to increase the reaction efficiency. qRT-PCR was performed using Fast SYBR^TM^ Green Master Mix (Thermo Fisher, Waltham, MA, United States), allowing the evaluation of the following genes from the intestine samples: monocarboxylate transporter 1 and 4 (MCT1, MCT4), sodium-coupled monocarboxylate transporter 1 (SMCT1) and GAPDH, used as an internal control. mRNA from samples of MRA were also analyzed by qRT-PCR, for quantification of Olfr78 and TGF-β gene expression, with β-actin as an internal control. Primer sequences are presented in Table 1. The mRNA relative quantification was calculated by the 2^-∆∆Ct^ method.

**TABLE 1:** Primer sequences used on qRT-PCR for analysis of proximal colon and mesenteric arteries.

Technique	Tissue	Gene	Sequences	
qRT-PCR	Proximal Colon		Forward primer	Reverse primer
		MCT1	GGT​GTC​ATT​GGA​GGT​CTT​GGG	GGT​GTC​ATT​GGA​GGT​CTT​GGG
		MCT4	TCA​GGA​GGC​AAG​CTG​CTG​GAC​GCA​A	AGT​TGC​CCA​GCA​GCA​GCA​CAA​GGG​A
		SMCT1	CTG​GGC​TTG​TTT​TCT​TTG​G	CGTTGTGCGTGCTGTTAC
		GAPDH	GGG​CAG​CCC​AGA​ACA​TCA​T	CCG​TTC​AGC​TCT​GGG​ATG​AC
	Mesenteric resistance arteries	Olfr78	ACA​GTC​CAA​ATC​GGC​ATG​GT	AGC​ACA​TTG​GAG​TGG​CAG​AA
		TGF-β	CCC​CAC​TGA​TAC​GCC​TGA​GT	AGC​CCT​GTA​TTC​CGT​CTC​CTT
		β-actin	AAG​ATT​TGG​CAC​CAC​ACT​TTC​TAC​A	CGGTGAGCAGCACAGGGT

Because of the reduced gene expression of the other SCFAs-sensing receptors on MRA samples, evaluation of GPR41, GPR43, GPR109a and β-actin, used as internal control, were performed by RT-PCR technique with the following amplification conditions: 8 min at 95°C; 40 cycles of 95°C for 30 s, 60°C for 30 s and 72°C for 1 min–all followed by a final step of 7 min at 72°C. Exclusively for β-actin, the number of cycles was reduced to 32 to avoid reaction saturation, maintaining all other conditions as described. Primer sequences are presented in Table 2. The amplification products were separated in an agarose gel (1%), and the band’s intensity was quantified with Image Lab (Bio-Rad, Hercules, CA, United States). Gene expression was obtained with normalization of the values from the receptor’s bands, by values from the internal control gene.

**TABLE 2:** Primer sequences used on RT-PCR for analysis of mesenteric arteries.

Technique	Tissue	Gene	Sequences	
RT-PCR	Mesenteric resistance arteries		Forward primer	Reverse primer
		GPR41	TCC​TCG​TGG​GAC​TAC​CCC​TC	CGG​CTT​GGA​ACT​TGG​AGG​AT
		GPR43	CTC​ACG​GGC​TTC​GGC​TTC​TA	CAC​CCC​TGT​CTG​TCT​TGG​TC
		GPR109a	AAG​ATC​TCC​AAC​CGG​ACG​GC	TTC​GAA​GGC​AAC​GGT​TGA​TG
		β-actin	GCG​AGT​ACA​ACC​TTC​TTG​CAG​C	CCG​TCT​CCG​GAG​TCC​ATC​AG

### 2.5 SCFA measurements

Fecal samples obtained from the cecum were weighed, crushed, and homogenized in 100 µl of distilled water. In 1.5 ml microtubes were added to the samples a 200 µl mixture of organic solvents composed of N-butanol, 40 µl HCl 0.1 mol/L, 10 mg citric acid and 20 mg sodium chloride, as described (Ribeiro et al., 2018). Samples were vigorously vortexed for 2 min and centrifuged at 13.000 x g at room temperature for 15 min. The supernatant was transferred to chromatographic vials and analyzed by gas chromatography. To quantify SCFAs, a calibration curve for the concentration range of 0.007–2 mg/ml was constructed, and measurements were performed following a published protocol (Ribeiro et al., 2018): chromatographic analyses were performed using a GC-2010 gas chromatograph (Shimadzu Instruments Inc., Kyoto, Japan), with a fused Ultra Stabilwax (Restec Corporation, United States) with dimensions of 30 m × 0, 25 mm internal diameter coated with a layer of polyethylene glycol 0.25 µm thick. The initial oven temperature was 100°C (hold 2 min), which was increased to 110°C at a rate of 15°C/min (hold 3 min). The FID temperature was maintained at 260°C, and the flow rates of H_2_, air, and the make-up gas N_2_ were 35, 350, and 25 ml/min, respectively. Sample volumes were injected at 260°C using a split ratio of approximately 25:1. Helium was used as the carrier gas at 1.00 ml/min. The runtime for each analysis was 11.95 min. Data are expressed as µmol/g feces.

### 2.6 Statistical analysis

All values are expressed as means ± SEM and ‘n’ describes the number of animals employed in the experiments. The results were analyzed using unpaired Student’s *t*-test or by two-way ANOVA, when appropriate. For correlation analyses of two variables, Pearson’s correlation coefficient (r) was used. When two-way ANOVA showed a significant statistical difference, Bonferroni’s post hoc test was applied to compare individual means, using GraphPad Prism Software 8.0 (GraphPad Software Copr., La Jolla, CA, United States). Differences were considered statistically significant at *p* <0.05 and are represented by ‘*’ in comparison to the Wistar group.

## 3 Results

As expected, SBP was higher in 6-months SHR than in Wistar rats (Supplementary Table S1). In addition, although the body weight was lower in SHR than in Wistar rats, higher left ventricle weight and left ventricle hypertrophy index (left ventricle to body weight ratio) were observed in SHR in comparison to Wistar rats (Supplementary Table S1), characterizing the hypertensive model used.

**FIGURE 1:**
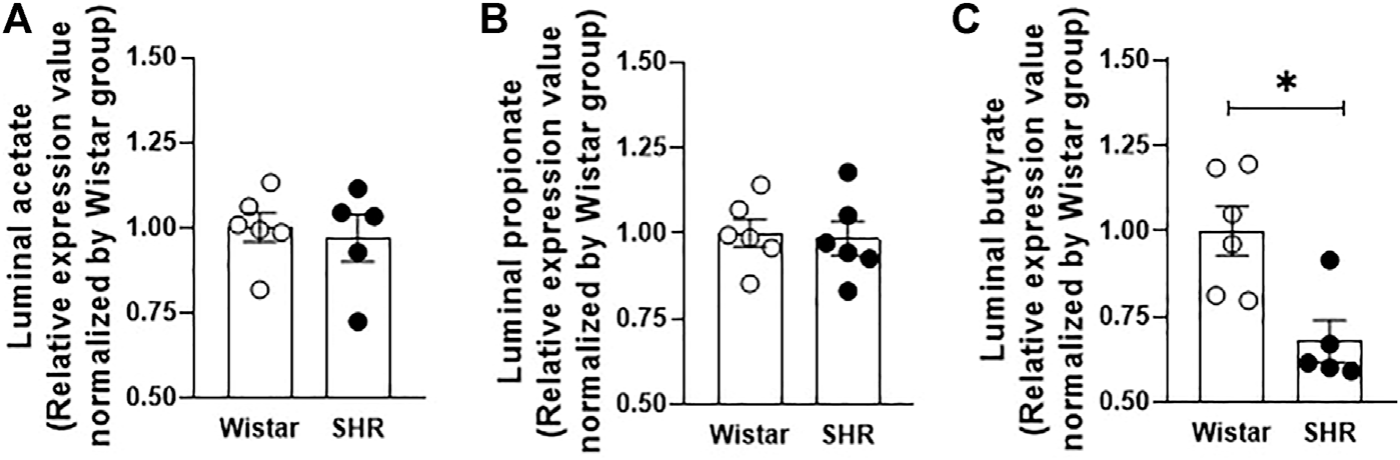
Reduced butyrate availability in cecum fecal content from Wistar and SHR. **(A)** acetate, **(B)** propionate and **(C)** butyrate concentrations were analyzed by gas chromatography. The number of animals used in each group is expressed in the graphic dots. Values are presented as the mean ± SEM and are normalized by the Wistar group. The statistical analysis was assessed by Student’s *t*-test: *p <0.05 vs Wistar.

**FIGURE 2:**
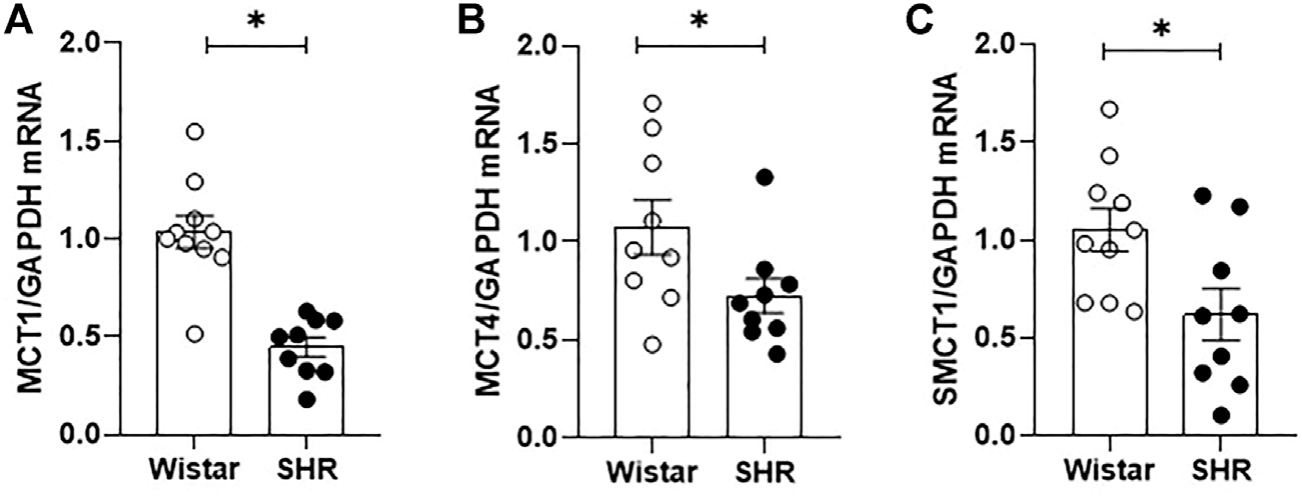
Impaired short chain fatty acids (SCFAs) intestinal absorption on adult SHR. Relative mRNA expression of **(A)** MCT1, **(B)** MCT4 and **(C)** SMCT1 transporters on proximal colon segments of adult Wistar and SHR, analyzed by qRT-PCR. The number of animals used in each group is expressed in the graphic dots. The mRNA relative quantification was calculated by the 2^-∆∆Ct^ method. Values are presented as the mean ± SEM. The statistical analysis was assessed by Student’s *t*-test: *p <0.05 vs Wistar.

Fecal samples obtained from rats’ cecum were analyzed by gas chromatography, allowing luminal SCFAs quantification. Figure 1 shows that whilst no changes in acetate and propionate luminal availability were observed between groups, butyrate cecum availability was lower in SHR, in comparison to Wistar rats. In addition, we also quantified by qRT-PCR the relative gene expression of three SCFA-symporters found in the intestinal epithelium. The MCT1, MCT4, and SMCT1 gene expressions were reduced (56%, 32%, and 41%, respectively) in proximal colon segments of SHR, when compared to the mRNA levels found on Wistar rats’ intestines (Figures 2A–C).

**FIGURE 3:**
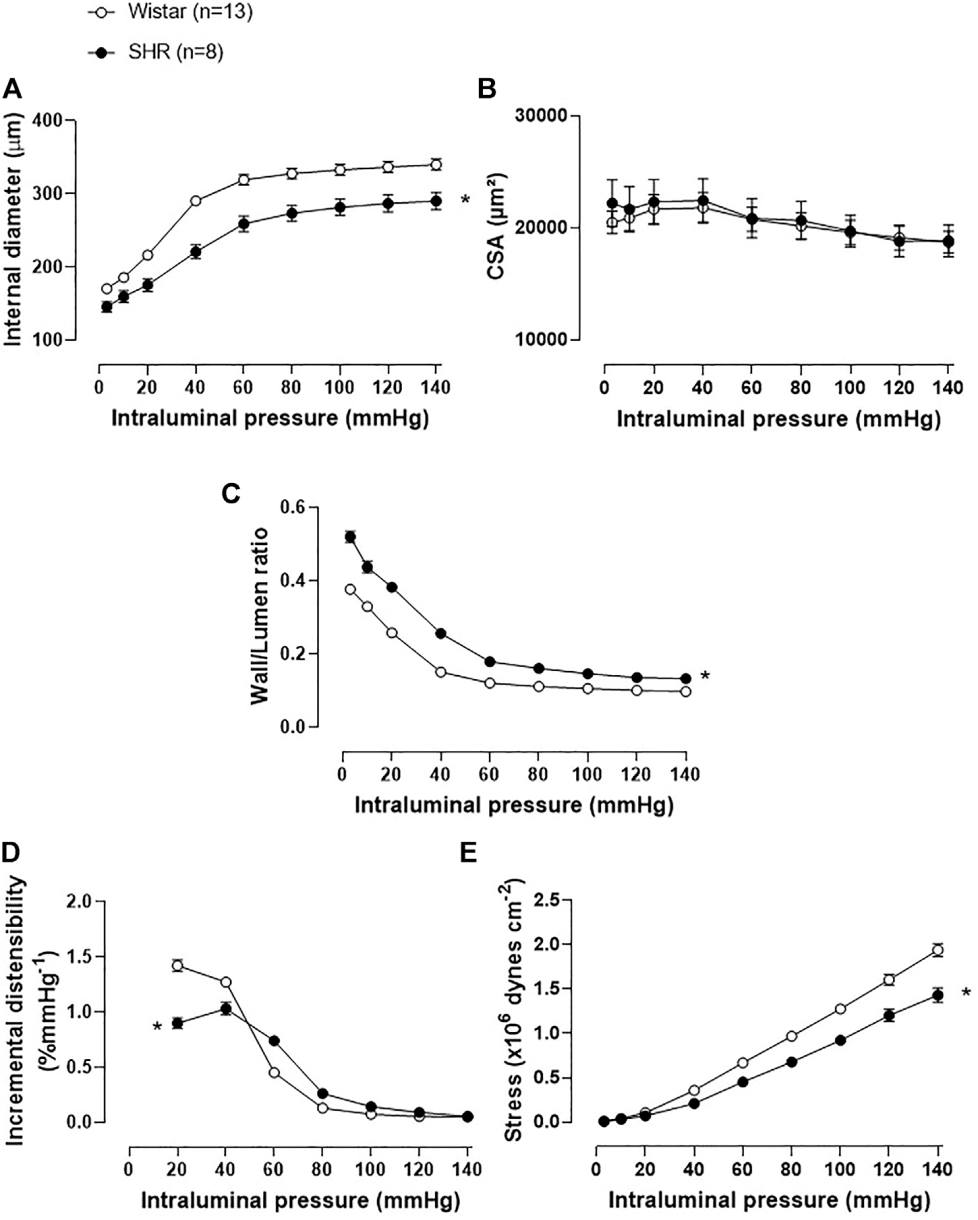
Structural and mechanical vascular remodeling on mesenteric resistance arteries of SHR. Comparison of the structural parameters: **(A)** internal diameter, **(B)** cross-section area (CSA), **(C)** wall/lumen ratio; and the mechanical parameters: **(D)** incremental distensibility and **(E)** stress of MRAs from adult Wistar and SHR. Analyses were obtained with a pressure myograph. The number of animals used in each experiment (n) is in parentheses. The results are expressed as the mean ± SEM and the statistical analysis was assessed by two-way ANOVA: *p < 0.05 vs Wistar.

Structural and mechanical properties of MRA were assessed in a pressure myograph, as shown in Figure 3. The internal diameter of MRA from the SHR was reduced when compared to the normotensive group (Figure 3A), while CSA was similar between groups (Figure 3B), characterizing an inward eutrophic remodeling in MRA of SHR. Moreover, the W/L ratio was increased in SHR MRAs (Figure 3C). Regarding the mechanical parameters, the incremental distensibility (Figure 3D) and circumferential wall stress (Figure 3E) were reduced in the MRA of SHR when compared to Wistar rats. The stress-strain curve of MRAs from SHR was displaced to the left as compared to the Wistar group (Supplementary Figure S1). β angle was assessed, as an index of arterial stiffness, showing augmented arterial stiffness in SHR MRAs (Supplementary Figure S1). As expected, higher mRNA expression of TGF-β, a cytokine involved in collagen expression, was observed in MRAs of SHR than in Wistar rats (Supplementary Figure S2).

**FIGURE 4:**
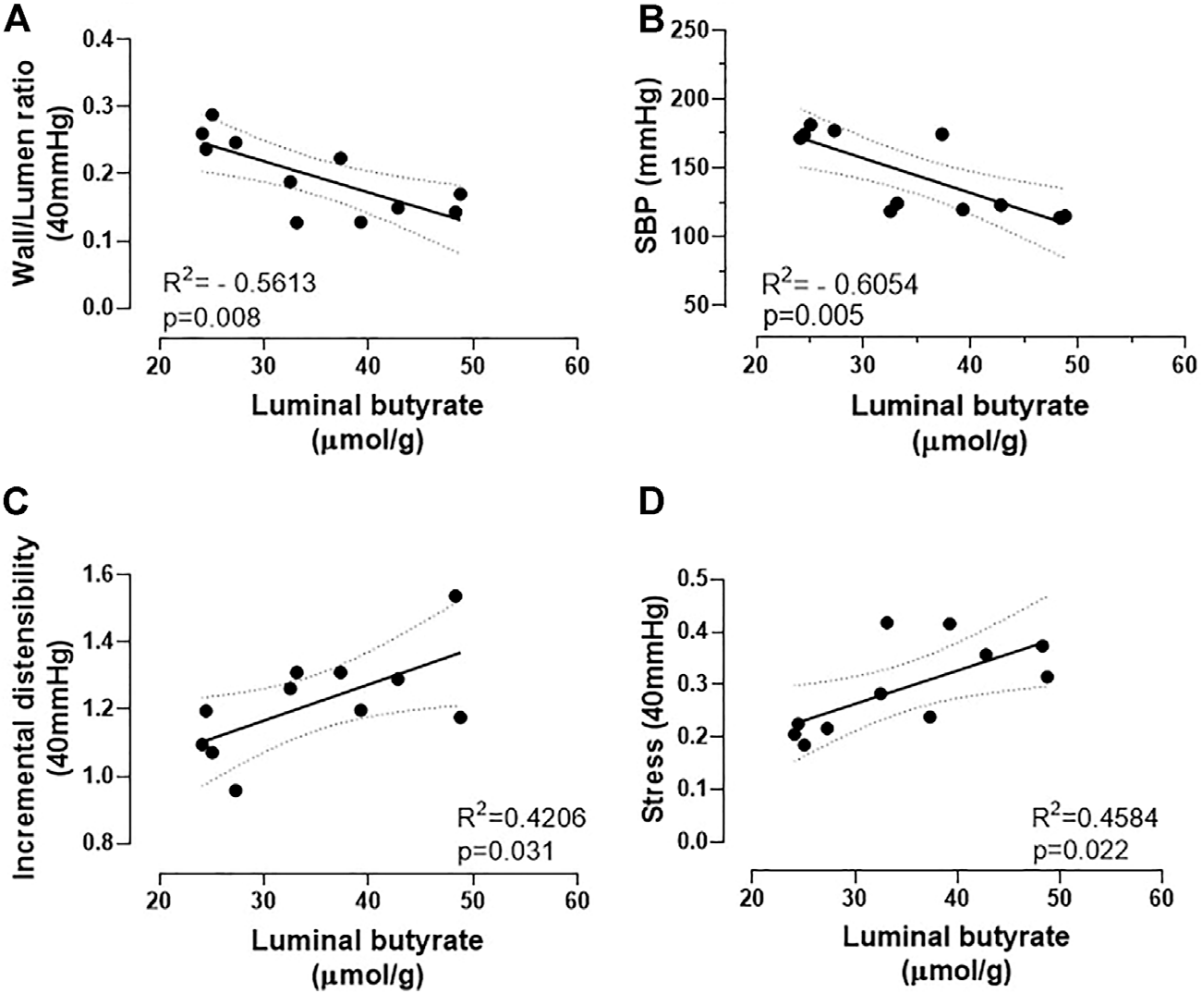
Correlations between structural and mechanical remodeling parameters of mesenteric resistance arteries or systolic blood pressure and intestinal butyrate availability. **(A)** negative correlation between wall/lumen ratio (a structural parameter) and **(B)** SBP with butyrate luminal content; **(C)** positive correlation between incremental distensibility and **(D)** stress with butyrate luminal content. Correlations were obtained by the Pearson correlation coefficient.

Therefore, for the first time, correlations were found between the structural and mechanical parameters described in the MRAs and the cecum butyrate availability. As shown in Figure 4A, the W/L ratio, a structural feature related to peripheral vascular resistance, presents a negative correlation with cecum butyrate content. In addition, a negative correlation between SBP and cecum butyrate availability was also observed (Figure 4B). Along with this data, both mechanical parameters studied (incremental distensibility and stress) showed positive correlations with cecum butyrate content (Figures 4C,D). Interestingly, TGF-β mRNA expression exhibited a positive correlation with the MRAs β angle, and β angle showed a negative correlation with the cecum butyrate content; but no significant correlation was observed between TGF-β mRNA and cecum butyrate content (Supplementary Figure S3 A–C).

**FIGURE 5:**
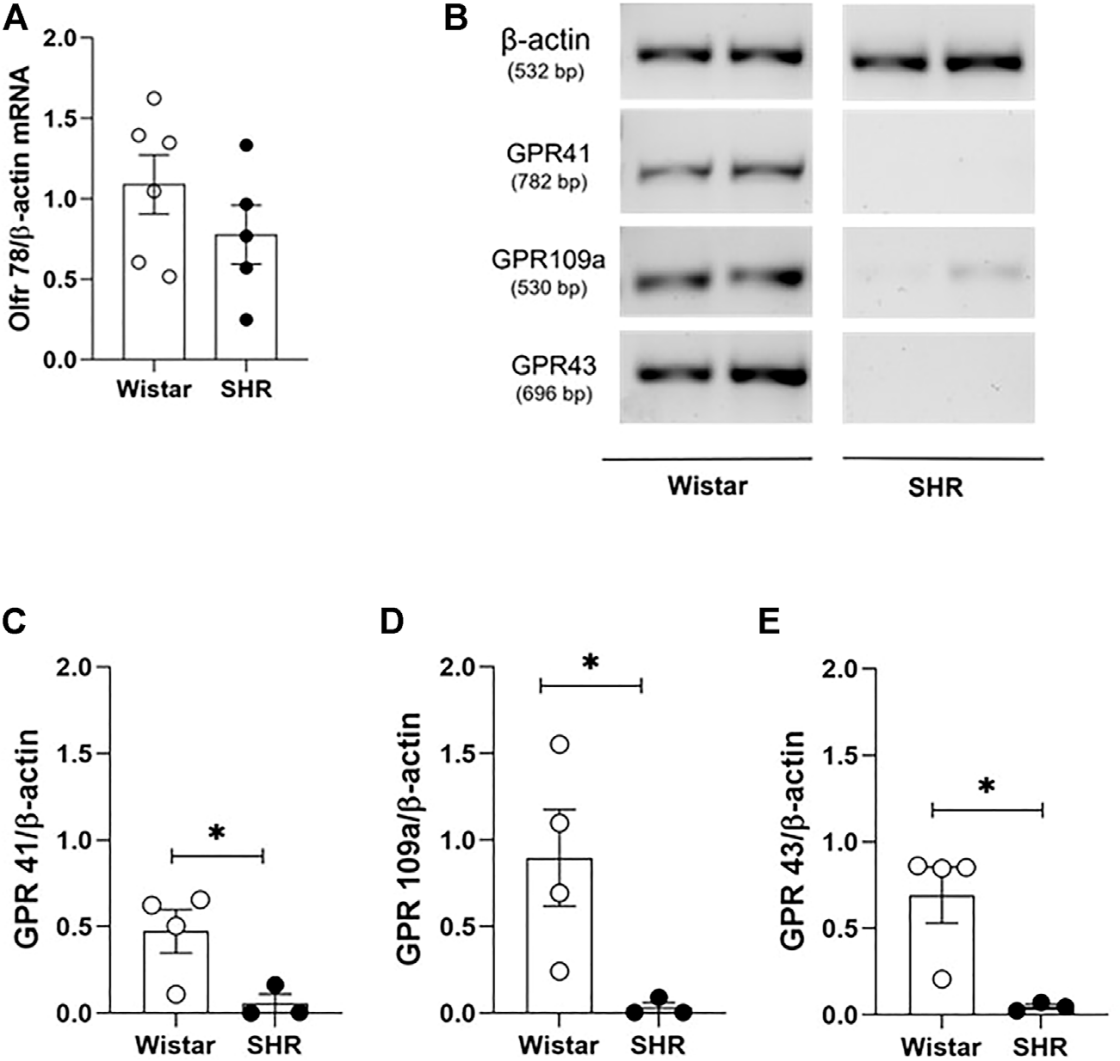
Reduced mRNA levels of short chain fatty acids (SCFAs)-sensing receptors on mesenteric resistance arteries of adult SHR. Relative receptor expression was evaluated by two different PCR approaches. **(A)** mRNA levels of Olfr78 receptor, analyzed by qRT-PCR; **(B)** representative results from RT-PCR analysis after evaluation on 1% agarose gel electrophoresis of **(C)** GPR41, **(D)** GPR109a and **(E)** GPR43 receptors present on mesenteric resistance arteries of adult Wistar and SHR. In the qRT-PCR result, mRNA relative quantification was calculated by the 2^-∆∆Ct^ method; whilst for RT-PCR analysis, gene expression was obtained with normalization of the values from the amplificated receptor’s bands, by values from the internal control. The number of animals used in each group is expressed in the graphic dots. Values are presented as the mean ± SEM. The statistical analysis was assessed by Student’s *t*-test: *p <0.05 vs Wistar.

To identify a possible pathway by which SCFAs availability and absorption could meddle with the structural and mechanical vascular remodeling in MRA of SHR, we evaluated mRNA levels of four SCFAs-sensing receptors, expressed on VSMCs or in the endothelium cells (Olfr78 (Pluznick et al., 2013; Mermer et al., 2021), GPR41/FFA3 (Natarajan et al., 2016), GPR43/FFA2 (Li et al., 2018) and GPR109a/HCA2 (Hughes-Large et al., 2014)). No difference was found in Olfr78 gene expression in MRA between groups (Figure 5A), but reduced GPR41, GPR109a, and GPR43 mRNA were detected on SHR MRAs when compared to the Wistar group (Figures 5C–E). These data suggest, for the first time, the existence of compromised signalling of SCFAs-sensing receptors on MRA of SHR.

## 4 Discussion

The most significant findings of this study are that butyrate availability correlates with the structural and mechanical remodeling of resistance arteries from SHR. The fecal butyrate content exhibited a negative correlation with the inward eutrophic remodeling, as well as a positive correlation with arterial stiffness, demonstrating that the lower availability of this molecule could favor vascular remodeling, promoting an increase in the peripheral vascular resistance, and compromising the prognosis of hypertension. The correlations described here might be the result of the reduced expression of SCFAs-transporters in the gut epithelium, and/or the decreased expression of the SCFAs-sensing receptors in the MRAs of SHR, which could both impair SCFAs intestinal absorption and its local signalling in the MRAs, compromising, therefore, vascular homeostasis.

It is well known that during the intense period of vascular and pressure changes observed during SHR growth, intestinal alterations are also present and are correlated with the development or maintenance of hypertension (Santisteban et al., 2017). At the age of 10 weeks, SHR has established intestinal microbiota dysbiosis, followed by intestinal pathology (Yang et al., 2015; Santisteban et al., 2017). Published studies report: (*i*) a higher Firmicutes/Bacteroidetes ratio; (*ii*) reduced small intestinal and colonic expression of tight junction proteins; (*iii*) enhanced intestinal epithelial permeability and (*iv*), increased intestinal fibrotic area and thickness of tunica muscularis layer (Yang et al., 2015; Santisteban et al., 2017). Additionally, as mentioned above, a reduction of SCFAs-producing bacteria/SCFAs availability was also described (Yang et al., 2015; Santisteban et al., 2017; Robles‐Vera et al., 2020; Yang et al., 2020). In line with these data, our study shows that fecal cecum samples from SHR had 32% less butyrate available when compared to normotensive rats, whilst no changes were found in acetate and propionate concentrations between strains. This reduced butyrate content is also in accordance with data already published, showing smaller amounts of circulating (Yang et al., 2019; Robles‐Vera et al., 2020) and fecal butyrate (Wu et al., 2019) availability in SHR. However, the equal amounts of acetate described here do not corroborate previous studies, which showed reduced acetate-producing bacteria and acetate availability levels on fecal samples from adult SHR (Yang et al., 2015; Robles‐Vera et al., 2020). This contradiction could be the result of a collection of fecal samples from different intestinal sites or could be a consequence of the distinct composition of the intestinal microbiota of hypertensive and normotensive lineages from different research centers – without ignoring the fact that the present study is the first to compare the SCFAs fecal composition between SHR and Wistar rats.

Also, it is important to report that we tried to evaluate the plasma concentration of SCFAs in our models. However, with the chosen method, the level of SCFAs in plasma was undetectable, a limitation of our study. Thus, we did not have enough information to determine if the butyrate content found in SHR cecum represented a lower bacteria production (Yang et al., 2015), or just a consequence of greater absorption of this compound by the intestinal epithelium in the hypertensive animals - a possibility that had already been hypothesized in humans studies (Verhaar et al., 2020). Therefore, we chose to analyze the relative expression of three important transporters present in the intestinal epithelium and responsible in large part for the transcellular absorption of luminal SCFAs by the host (Sivaprakasam et al., 2017; Stumpff, 2018; Dalile et al., 2019). We observed reduced expression of MCT1, MCT4, and SMCT1 in the SHR intestinal epithelium. MCT1 and MCT4 are both H^+^:monocarboxylate electroneutral cotransporters, the first being expressed in the apical membrane and the last in the basolateral membrane of the epithelial cells, allowing not only the absorption of butyrate, but also the transport of other aliphatic short-chain molecules as acetate, propionate, lactate, and pyruvate - depending on the transmembrane concentration gradient (Sivaprakasam et al., 2017; Stumpff, 2018; Dalile et al., 2019). SMCT1 is a sodium-coupled electrogenic transporter expressed in the apical membrane of colonic epithelial cells which concede the entrance of two sodium ions and any SCFA molecule available (Sivaprakasam et al., 2017; Stumpff, 2018). The minor presence of MCT1 and MCT4 transporters in SHR intestinal epithelium had already been described by other groups (Yang et al., 2019; Robles‐Vera et al., 2020), but we are the first to also identify reduced SMCT1 mRNA levels in this model of hypertension, demonstrating a limitation in the absorption of SCFAs by the reduced expression of three, out of the five, known direct intestinal SCFAs transporters (Dalile et al., 2019).

As butyrate can directly regulate the expression of those transporters evaluated (Borthakur et al., 2008; Ziegler et al., 2016) by, among other mechanisms, the inhibition of HDAC activity (Sivaprakasam et al., 2017), we could suggest by the reduced expression of MCT1, MCT4, and SMCT1, that our data shows a small butyrate production instead of higher absorption of this SCFA. Thus, we can conclude that epithelium absorption of all SCFAs is compromised in the proximal colon of SHR, interfering not only with the butyrate host absorption, but also with acetate and propionate transport, even though the availability of both these SCFAs were not reduced in the fecal cecum samples from SHR.

To correlate butyrate availability with resistance artery remodeling, MRAs structure and mechanical parameters were evaluated in a pressure myograph, showing an inward eutrophic remodeling and an enhanced W/L ratio on SHR MRAs, as previously observed (Briones et al., 2003; Roque et al., 2013). In addition, we also described reduced incremental distensibility and circumferential wall stress, in accordance with higher β angle values in MRAs of SHR, indicating the stiffness of those arteries (Briones et al., 2003; Roque et al., 2013). TGF-β, considered a profibrotic cytokine related to collagen up-regulation, had higher gene expression in MRAs of SHR and a positive correlation with the β values, suggesting an important pro-fibrotic role in the mechanical remodeling delineated. Using these structural and mechanical parameters obtained, we described for the first-time correlations between MRA’s structural and mechanical remodeling and intestinal butyrate availability. W/L ratio, an index of peripheral vascular resistance, presented a negative correlation with cecum butyrate content, indicating that the lower the availability of butyrate is, the greater the vascular resistance imposed by the arterial bed. Interestingly, in line with this correlation, we also describe a negative correlation between SBP and luminal butyrate availability, showing that the lower the content of cecum luminal butyrate available, the higher is the blood pressure found. Furthermore, arterial distensibility, circumferential stress and β value—mechanical parameters evaluated - showed significative correlations with the cecum butyrate content, allowing us to conclude that the lower the amount of this SCFA is, the smaller will be the arterial distensibility and the circumferential wall stress developed and higher will be the stiffness. However, drawing attention to the complexity of the mechanical remodeling process, TGF-β gene expression did not exhibit a correlation with the cecum butyrate content, suggesting that maybe collagen is not the elastic passive vascular component influenced by the SCFAs. Unfortunately, we were unable to assess the other passive components of the MRAs, which is another limitation of our study.

Nevertheless, as the vascular remodeling is an intricate and active process (Mulvany, 2002; Masi et al., 2020), it is difficult to characterize a unique route in which the reduced butyrate content could influence vascular smooth muscle cells (VSMCs) and vessel wall elastic content reorganization. It could happen by the regulation of vascular tonus through the activation of SCFAs-sensing receptors (Pluznick et al., 2013; Natarajan et al., 2016; Onyszkiewicz et al., 2019; Poll et al., 2020) or PPAR isoforms (Tian et al., 2021); by reducing the production or availability of ROS (Mathew et al., 2014; Gómez-Guzmán et al., 2015; Robles‐Vera et al., 2020; Tian et al., 2021); decreasing vascular inflammation (Bartolomaeus et al., 2019; Robles‐Vera et al., 2020), and even controlling VSMCs proliferation and migration (Ho et al., 2015; Mathew et al., 2019). With that in mind, we quantified mRNA levels of SCFAs-sensing receptors on MRAs, trying to find a pathway to corroborate the correlations described.

We found that Olfr78, a receptor expressed on VSMCs (Pluznick et al., 2013; Mermer et al., 2021), showed no difference in relative expression between groups. However, GPR41 (present in the vascular endothelium (Natarajan et al., 2016)), GPR43, and GPR109a (Poll et al., 2020) were reduced on SHR MRAs when compared to the control group. A reduction of these receptors was already described in the gut (Toral et al., 2019a) and brain paraventricular nucleus of SHR (Toral et al., 2019a; Yang et al., 2019); but to our knowledge, changes in SCFAs-sensing receptors on SHR MRAs were not yet described until the present moment.

Olfr78 role in hypertension was characterized primarily in the renal afferent arterioles, where it was related to the release of renin after stimulus with propionate (Pluznick et al., 2013; Poll et al., 2021). Since it is a receptor coupled to a Gs protein and expressed in the VSMCs (Pluznick et al., 2013; Poll et al., 2020; Mermer et al., 2021), its local activation may likely be related to vascular muscle relaxation in the presence of acetate or propionate. Nevertheless, this vascular signalling has not yet been demonstrated in the literature. On the other hand, GPR41 and GPR43 are both present in vessels, being receptors coupled to Gi and Gi and Gq proteins, respectively; but only the former was described as expressed in the endothelium cells (Natarajan et al., 2016; Poll et al., 2020). The stronger ligand for these receptors is propionate, but they also could be activated by butyrate and acetate in the µmol/L range (Poll et al., 2020). Interestingly, GPR41 KO mice developed isolated systolic hypertension at 3 months of age; presented increased pulse wave velocity at 6 months, and had higher collagen area and elastin fraction in the aorta, when compared to control mice, suggesting a correlation between the absence of this receptor and the development of mechanical vascular changes in a conductance artery (Natarajan et al., 2016). Notwithstanding, little is known about GPR109a presence and function in vessels (Poll et al., 2020). It was suggested that this receptor is expressed on human endothelial aortic cells and in microvascular endothelial cell culture, improving angiogenic function during exposure to pathophysiological concentrations of saturated fatty acids *in vitro,* in response to nicotinic acid (Hughes-Large et al., 2014). Altogether, this data suggests that a reduction in SCFAs-sensing receptors expression on MRAs could harm vascular homeostasis in SHR, on top of the SCFAs compromised intestinal absorption and reduced luminal butyrate availability. However, it is not yet known if these inefficient SCFAs signalling would affect vascular homeostasis, and even if it corroborates vascular remodeling - but these points are the purpose of other studies under development in our research group.

In conclusion, the present study described the existence of impaired intestinal butyrate production and absorption in adult SHR, which correlates to vascular remodeling and blood pressure values. In this way, it is possible to suggest that the reduced butyrate content and impaired SCFAs signalling found are correlated with structural and mechanical vascular adjustments in resistance arteries, influencing peripheral vascular resistance and the maintenance of hypertension. The described data brings new evidence on the role of SCFAs production, absorption, and SCFAs-sensing receptors signalling as therapeutic targets for hypertension and end-organ damage.

## Data Availability

The raw data supporting the conclusion of this article will be made available by the authors, without undue reservation.

## Abbreviations

CSA, Cross section area; De, External diameter; Di, Internal diameter; HDAC, Histone deacetylases; KHS, Krebs-henseleit solution; MCT1, Monocarboxylate transporter 1; MCT4, Monocarboxylate transporter 4; MRAs, Mesenteric resistance arteries; PPAR, Peroxisome proliferator-activated receptors; ROS, Reactive oxygen species; SBP, Systolic blood pressure; SCFAs, Short-chain fatty acids; SHR, Spontaneously hypertensive rats; SMCT1, Sodium coupled monocarboxylate transporter 1; TGF-β, Transforming growth factor β; VSMC, Vascular smooth muscle cells; W/L, Wall/lumen ratio; WT, Wall thickness.
