# Supplementary material for: Reduced intestinal butyrate availability is associated with the vascular remodeling in resistance arteries of hypertensive rats
Source: Front Physiol. 2022 Sep 29;13:998362. doi: 10.3389/fphys.2022.998362 (PMC9558208; doi:10.3389/fphys.2022.998362)

Reduced intestinal butyrate availability is associated with the vascular remodeling in resistance arteries of hypertensive rats

Patrizia Dardi^1^, Rosangela Aparecida dos Santos Eichler^2^, Sarah de Oliveira^3^, Marco Aurélio Ramirez Vinolo^3^, Niels Olsen Saraiva Câmara^4^ and Luciana Venturini Rossoni^1^*

**Supplementary data**

**Supplementary table 1** – Hemodynamic and morphometric parameters of the adult Wistar and SHR.

SBP, systolic blood pressure; BW, body weight; LV, left ventricle; LV/BW, ratio of left ventricle weight to body weight. The results are expressed as the mean ± SEM. The number of animals used in each evaluation (n) is in parentheses. Statistical analysis was assessed by Student *t*-test. *p<0.05 *vs*. Wistar.

|  | **Wistar** | **SHR** |
| --- | --- | --- |
| **SBP (mmHg)** | 122 ± 1.51 (n=18) | 179 ± 1.85 * (n=15) |
| **BW (g)** | 463 ± 9.91 (n=16) | 329 ± 6.30 * (n=15) |
| **LV (mg)** | 819 ± 21.8 (n=16) | 942 ± 28.4 * (n=15) |
| **LV/BW (mg/g)** | 1.77 ± 0.04 (n=16) | 2.86 ± 0.08 * (n=15) |


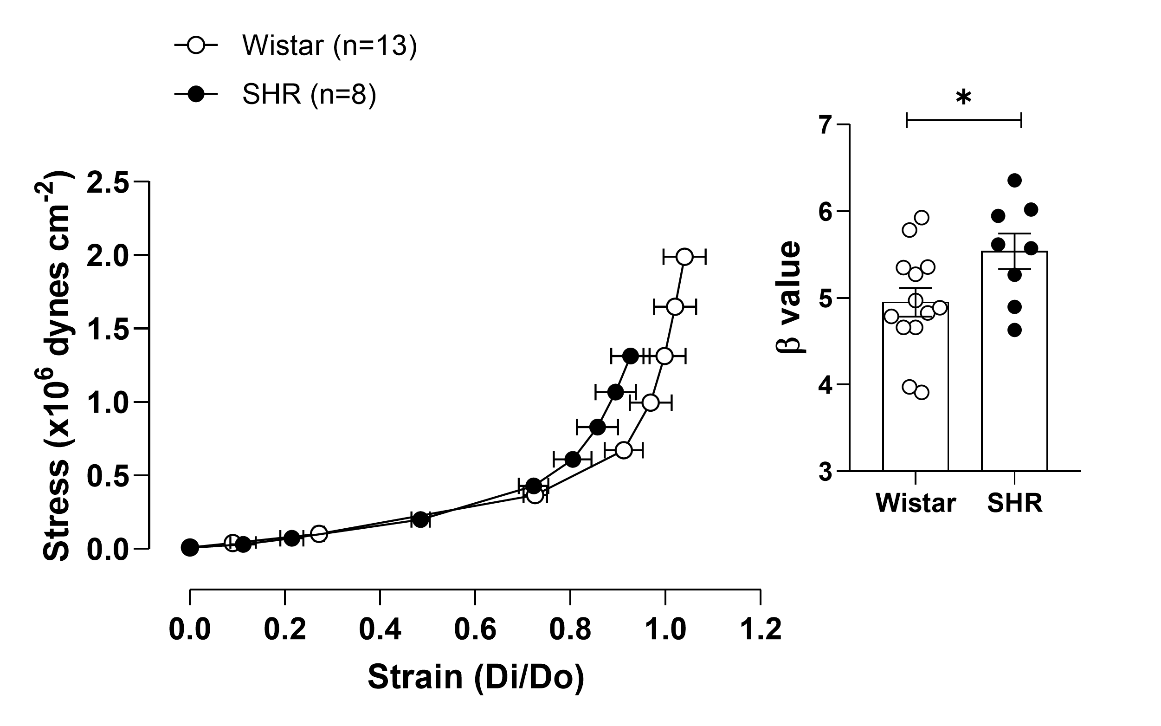


**Supplementary figure 1 – Arterial stiffness evaluation on stress-strain curves of Wistar and SHR mesenteric resistance arteries.** Arterial stiffness was assessed through the *Young elastic modulus* on the stress-strain curve of mesenteric resistance arteries from adult Wistar and SHR. The tangential elastic modulus was determined, providing the β angle – a direct index of arterial stiffness (right graph). The number of animals used in each group is expressed in parentheses (n) or in the graphic dots. Values are presented as the mean ± SEM. The statistical analysis was assessed by Student’s t-test: *p<0.05 vs. Wistar.

**Supplementary figure 2 – Enhanced mRNA levels of TGF-β on mesenteric resistance arteries of SHR.** Relative TGF-β expression was evaluated by qRT-PCR on mesenteric resistance arteries of adult Wistar and SHR. mRNA relative quantification was calculated by the 2^-∆∆Ct^ method. The number of animals used in each group is expressed in the graphic dots. Values are presented as the mean ± SEM. The statistical analysis was assessed by Student’s *t*-test: *p<0.05 vs. Wistar.


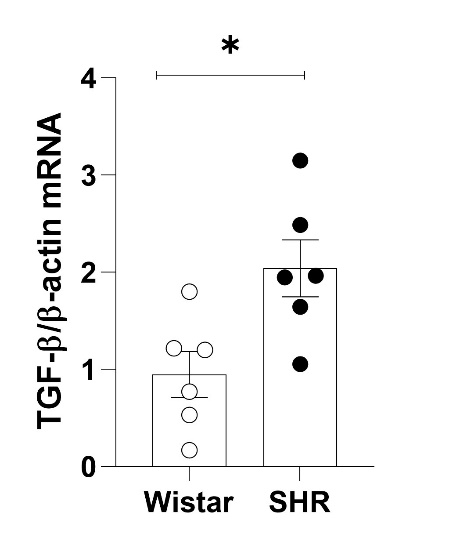


**Supplementary figure 3 – Correlations between TGF-β mRNA levels and β value or intestinal butyrate availability and between β value and intestinal butyrate availability. A)** positive correlation between arterial gene expression of TGF-β and β value (an arterial stiffness index); **B)** the absence of correlation between TGF-β gene expression and butyrate cecum luminal content; and **C)** negative correlation between β value and butyrate cecum luminal content. Correlation was obtained by the Pearson correlation coefficient.


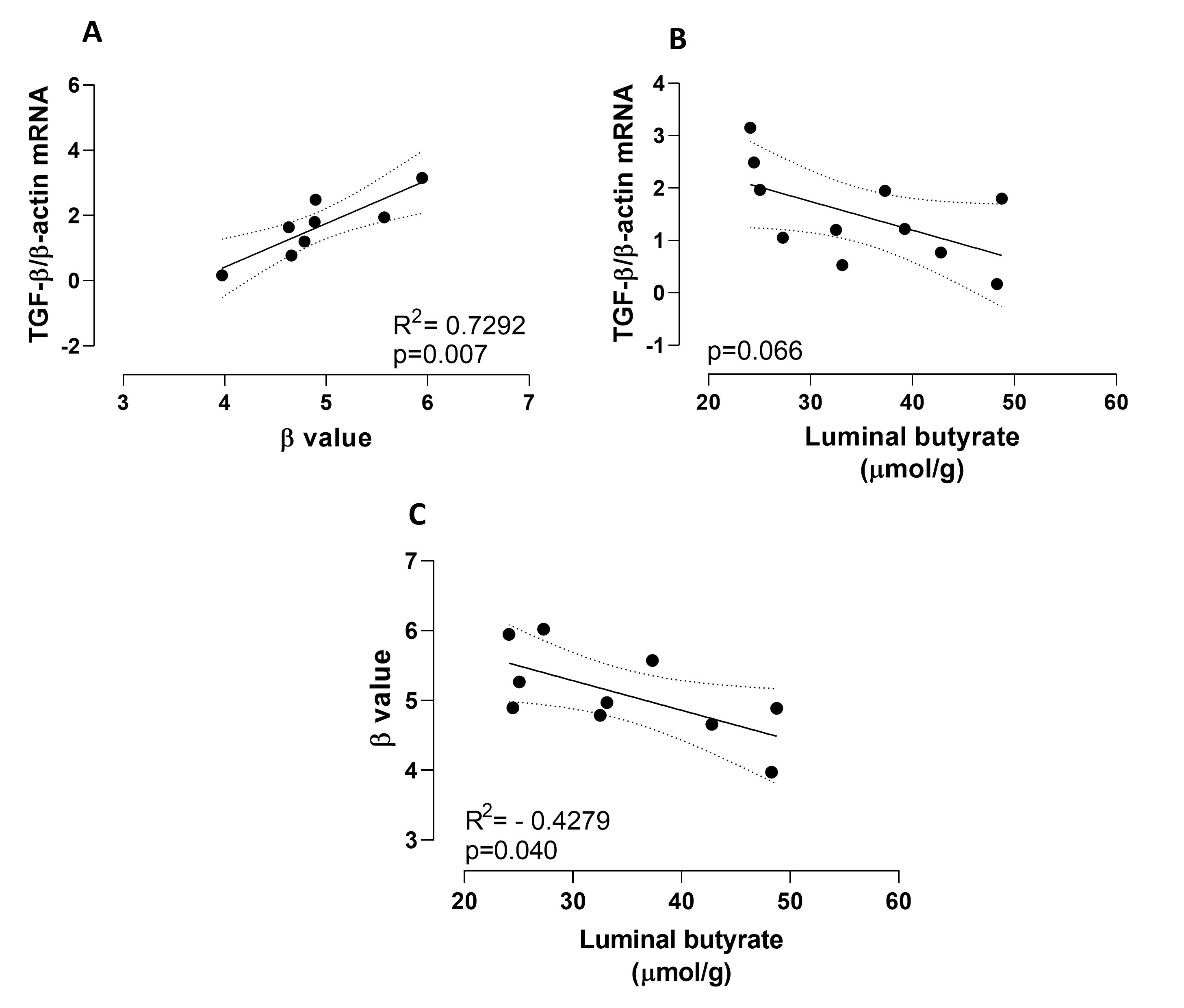

Supplement: Supplementary file 1 [file DataSheet1.docx]
